# Grand-maternal lifestyle during pregnancy and body mass index in adolescence and young adulthood: an intergenerational cohort study

**DOI:** 10.1038/s41598-020-71461-5

**Published:** 2020-09-02

**Authors:** Ming Ding, Susanne Strohmaier, Eva Schernhammer, Changzheng Yuan, Qi Sun, Karin B. Michels, Rulla Tamimi, Jorge E. Chavarro

**Affiliations:** 1 Department of Nutrition, Harvard School of Public Health, Harvard University, 655 Huntington Ave, Boston, MA 02115 USA; 2 Institute of Clinical Biometrics, Center for Medical Statistics, Informatics, and Intelligent Systems, Medical University of Vienna, Vienna, Austria; 3 Department of Epidemiology, Center for Public Health, Medical University of Vienna, Vienna, Austria; 4 Channing Division of Network Medicine, Department of Medicine, Brigham and Women’s Hospital and Harvard Medical School, Harvard University, Boston, MA USA; 5 School of Public Health, Zhejiang University, Hangzhou, Zhejiang China; 6 Department of Epidemiology, Fielding School of Public Health, University of California, Los Angeles, USA; 7 Division of Epidemiology, Department of Healthcare Policy and Research Weill Cornell Medicine, New York, USA; 8 Department of Epidemiology, Harvard School of Public Health, Harvard University, Boston, MA USA

**Keywords:** Diseases, Medical research, Risk factors

## Abstract

To examine associations of healthy lifestyle during pregnancy with body mass index (BMI) and risk of overweight or obesity of grandchildren during adolescence and young adulthood. Our study population included 14,001 grandmother–mother–child triads comprised of participants of two ongoing prospective cohort studies of related individuals. We used self-reported grand-maternal gestational weight gain, diet, physical activity, and smoking during pregnancy to create a lifestyle score ranged from 0 to 12, with a higher score indicating healthier lifestyle. Grandchild BMI was self-assessed in follow-up questionnaires. Compared with individuals whose grandmothers had the least healthy lifestyle during pregnancy, individuals whose grandmothers had the most healthy lifestyle had 0.17 (95% CI 0.01, 0.33; P for trend = 0.05) kg/m^2^ lower BMI and 7% (95% CI 2%, 12%; P for trend = 0.001) lower risk of overweight or obesity during adolescence and young adulthood. The inverse associations between grand-maternal lifestyle and BMI in grandchildren were mainly mediated by maternal pre-pregnancy BMI (mediation effect: 64%; P value = 0.001). Overall, maternal BMI, along with maternal socioeconomic status and lifestyle factors in the second and third generations accounted for all of the inter-generational association (mediation effect: 99%; P value < 0.001). The inverse associations of grand-maternal lifestyle with BMI of offspring were not modified by grand-maternal pre-pregnancy BMI, grandchild age, or grandchild gender. Grandchildren of women who had the healthiest lifestyles during pregnancy defined by no excess gestational weight gain, no smoking, a healthy diet and being physically active, were less likely to be overweight or obese in adolescence and early adulthood.

## Introduction

In the past decades, the prevalence of obesity in children has escalated and become an important public health problem worldwide. Globally, an estimated 43 million children under age 5 were overweight or obese in 2010^1^. If this trend continues, nearly 60 million children under age 5 are projected to be overweight or obese by 2020^1,2^. Childhood obesity can cause a wide range of cardiovascular, endocrine, gastrointestinal, and pulmonary complications^3^. Moreover, compared to normal weight children, overweight or obese children have a higher rate of being overweight or obese as adults and experience higher risks of cardiometabolic diseases and cancer later in life^4,5^.

Childhood obesity originates early in life. Prospective cohort studies have shown that maternal lifestyle factors such as weight gain and smoking during pregnancy were associated with higher risk of obesity in the offspring^6–11^. Some randomized controlled trials (RCT) have shown that diet and exercise during pregnancy prevents excessive gestational weight gain^12,13^. By combining individual lifestyle factors, maternal adherence to a healthier pre-pregnancy lifestyle including high-quality diet, adequate physical activity, normal body mass index (BMI), and no smoking was associated with lower risk of obesity in the offspring^14,15^. However, few studies have examined whether the association for maternal lifestyle during pregnancy persists to the third generation.

In this study, we examined the association of grand-maternal lifestyle during pregnancy with BMI and risk of overweight or obesity of offspring by adopting a three-generation study design. This unique design included 14,001 grandmother–mother–child triads comprised of participants in the Nurses’ Mother Cohort (F1), the Nurses’ Health Study II (NHS II, F2), and the Growing Up Today Study (GUTS, F3).

## Methods

### Study population

NHS-II was established in 1989 when 116,430 female nurses completed a questionnaire about lifestyle factors, anthropometric variables and disease prevalence. Follow-up questionnaires are sent biennially to collect updated information. Food frequency questionnaires were initially sent in 1991 and every four years thereafter. In 2001, 35,794 mothers of NHS-II participants were invited to complete a questionnaire regarding their pregnancy with their NHS-II participant daughter. Details about the Nurses’ Mothers’ Cohort have been published elsewhere^16^.

GUTS participants are offspring of NHSII participants who had at least one child between the ages of 9 and 14 years and consented to their children’s enrollment. In 1996, an invitation letter and the baseline questionnaire were then sent to 25,000 children, and approximately 68% if the girls (n = 9,039) and 58% of the boys (n = 7,843) returned a completed questionnaire (total n = 16,882). In 2004, GUTS was extended to include an additional 10,923 children aged 9–14 years. Participants have been followed-up with self-administered follow-up questionnaires administered every 1–3 years.

After merging the Nurses’ Mothers’ Cohort, NHS-II, and GUTS, our study included 14,001grandmother–mother–child triads that grandmother (F1) and offspring (F3) were both enrolled and have available information on grand-maternal lifestyle factors during pregnancy and body weight in the offspring. Our study was approved by the Institutional Review Boards of the Harvard School of Public Health and Brigham and Women’s Hospital. For participants under 18 years, consent from parents or guardians were obtained. Return of a completed questionnaire was considered as informed consent in these cohort studies. All methods were performed in accordance with the relevant guidelines and regulations.

### Assessment of lifestyle factors in grandmothers

In 2001, mothers of NHS-II participants (F1) were administered a questionnaire asking about gestational weight gain, smoking status, physical activity, and diet during pregnancy. Weight gain during pregnancy were asked in categories, including < 10 lbs, 10–14 lbs, 15–19 lbs, 20–29 lbs, 30–40 lbs, and 40 + lbs. For smoking status, grandmothers were asked whether they smoked during pregnancy, during which trimester they smoked, and the number of cigarettes per day*.* Grandmothers were asked about physical activity patterns at work, home, and other places in categories, including highly active (equivalent to walking about 3 or more miles every day), somewhat active (equivalent to walking about 2 miles every day), active (equivalent to walking about 1 miles every day), mostly inactive (equivalent to walking about half a mile or less every day), and inactive (no walking or other regular exercise). Diet was assessed using a 24-item food frequency questionnaire. In the food frequency questionnaire, grandmothers were asked how often on average they consumed a standard portion size of each food item during pregnancy. We grouped the foods into five groups, namely red and processed meat, whole grain, vegetables, fruits and fish. We defined a healthy diet as high intakes of whole grain, vegetables, fruits, and fish and low intake of red and processed meat, and derived a diet quality score, with a higher score indicating a healthier diet. We assigned scores of 1–3 to each of the four factors according to adherence to pre-specified standards^6,17–19^, with a higher score indicating a healthier lifestyle (Table 1). Specifically, the most healthy lifestyle was defined as gestational weight gain within the 2009 IOM guidelines, no smoking, most healthy diet, and moderate physical activity; and the least healthy lifestyle was defined as gestational weight gain outside of guidelines, heavy smoking, least healthy diet, and low activity. We summed the score of each lifestyle factor to obtain a total score (range 4–12). The assumption of summing the score was that all lifestyle factors are equally weighted and have the same importance in associating with offspring body weight; and the association of the score with offspring BMI was linear. We categorized the total score into four groups (≤ 7, 8, 9, and 10–12) of approximately equal size. We excluded participants with more than two factors missing given that limited information on grandmaternal lifestyle was provided (n = 31).

**Table 1:** Assignment of grand-maternal lifestyle score (F1) during pregnancy based on grand-maternal weight gain, smoking, diet quality, and physical activity.

Scored factor	Score	Scoring criteria	Scoring standard
Weight gain	1: Gestational weight gain outside of guidelines 3: Gestational weight gain within IOM guidelines	(1) Pre-pregnancy BMI < 25 kg/m^2^ and weight gain < 20 or > 40 lb during pregnancy (2) Pre-pregnancy BMI 25–30 kg/m^2^ and weight gain < 15 or > 30 lb during pregnancy (3) Pre-pregnancy BMI ≥ 30 kg/m^2^ and weight gain < 10 or > 20 lb during pregnancy (1) Pre-pregnancy BMI < 25 kg/m^2^ and weight gain 20–40 lb during pregnancy (2) Pre-pregnancy BMI 25–30 kg/m^2^ and weight gain 15–30 lb during pregnancy (3) Pre-pregnancy BMI ≥ 30 kg/m^2^ and weight gain 10–20 lb during pregnancy	Institute of Medicine (IOM) guidelines in 2009^17^
Smoking	1: Heavy smoking 2: Moderate smoking 3: No smoking	Grandmother smoked > 14 cigarettes/day during all three trimesters of pregnancy Grandmother smoked during the first two trimesters or ≤ 14 cigarettes/day in all three trimesters of pregnancy Grandmother did not smoke during pregnancy	Ding et al. 2017^6^
Diet quality score	1: Least healthy diet 2: Less healthy diet 3: Most healthy diet	Lowest tertile of diet quality score featured by low intake of meat and high intakes of vegetables, fruits, fish, and whole grains during pregnancy Middle tertile of diet quality score Highest tertile of diet quality score	The 2015–2020 Dietary Guidelines for Americans for Pregnant Women^18^
Physical activity	1: Low activity 2: Intense physical activity 3: Moderate physical activity	Lowest tertile of self-reported physical activity during pregnancy Highest tertile of self-reported physical activity during pregnancy Middle tertile of self-reported physical activity during pregnancy	2008 Physical Activity Guidelines for Americans, Key Guidelines for Women During Pregnancy^19^

A validation Study was conducted among 146 participants in the Nurses’ Mothers’ Cohort, who were also participants in the National Collaborative Perinatal Project (NCPP)^20^. Reproducibility of grand-maternal recall of pregnancy-related events was assessed by administering same questionnaires to the mothers two years apart, and validity was assessed by comparing the grand-maternal recall to the NCPP record. The validity of recalled grand-maternal smoking was high (sensitivity = 0.86, specificity = 0.94), and the validity of gestational weight gain was acceptable (correlation coefficient = 0.42)^20^.

### Outcome assessment

GUTS participants (F3) reported their height and weight at baseline, and updated these data on follow-up questionnaires. Adolescents have been found to be able to provide valid reports of height and weight^21–23^. Body mass index (BMI) was calculated as the ratio of weight (kg) to height squared (m^2^). In adolescents (< 18 years), overweight or obesity was defined as a BMI at or above the age- and sex-specific cutoffs proposed by the International Obesity Task Force (IOTF)^24^. In adults (≥ 18 years), overweight/obesity was defined as BMI ≥ 25 kg/m^2^. We identified a participant as overweight if the person was overweight at any time of assessment.

### Assessment of covariates and mediators

The mothers of NHS-II participants (F1) reported gestational age, age at birth, level of education, and pre-pregnancy BMI by questionnaire. The NHS-II participants (F2) reported BMI, smoking status, amount of physical activity, and income at baseline and every 2 years thereafter. Diet was assessed by a 131-item food frequency questionnaire in 1991 and every four years thereafter, and an alternative healthy eating index has been derived as an indicator of diet quality which was described elsewhere^25^. We collected information on smoking status, diet, and physical activity in GUTS (F3) at baseline and every 2–4 years thereafter. The KidMed Index has been derived in the GUTS participants as a measure of diet quality^26^. For the analysis assessing mediation effect of grand-maternal lifestyle on BMI of offspring, we used lifestyle factors measured closest before the birth date of the offspring in the NHSII and lifestyle factors measured at baseline in the GUTS as potential mediators.

### Statistical analysis

We used generalized estimating equations to account for the non-independence of sibling clusters to assess associations of grand-maternal lifestyle during pregnancy with BMI and risk of overweight or obesity of grandchildren, adjusting for gestational age, age at birth, level of education, and pre-pregnancy BMI. As to associations between individual grand-maternal lifestyle factors and offspring BMI, all lifestyle factors are mutually adjusted for each other. We examined whether F2 and F3 lifestyle factors and F2 socioeconomic status singly or jointly mediated the associations between grand-maternal lifestyle factors and BMI of offspring. Mediation effect was evaluated using the method proposed by Lin et al.^27^. Specifically, mediation effect was calculated as difference between coefficients with and without adjusting for mediator. Proportion of mediation was calculated as mediation effect over coefficients without adjusting for mediator. The assumptions of mediation analysis include no reverse causal effects of outcome on mediator; no measurement error in the mediator; and no confounding between mediator and outcome. Mediation analysis also makes all of the standard assumptions of the general linear model (i.e., linearity, normality, homogeneity of error variance, and independence of errors). We examined effect modification by F1 pre-pregnancy BMI, F3 age, and F3 sex by adding interaction terms to the model. All tests of statistical significance were two sided, with P value < 0.05 denoting significant findings. All statistical analyses were performed using SAS Version 9.2 (SAS Institute Inc., Cary, NC, USA).

### Patient involvement

No patients were involved in setting the research question or the outcome measures, nor were they involved in developing plans for design or implementation of the study. No patients were asked to advise on interpretation or writing up of results. There are no plans to disseminate the results of the research to study participants or the relevant patient community.

### Ethical approval

The study protocol was approved by the institutional review boards of the Brigham and Women’s Hospital and the Harvard TH Chan School of Public Health. The completion of the self administered questionnaire was considered to imply informed consent.

## Results

Our study included 14,001 GUTS participants (F3) paired to their 9,157 mothers (F2) and grandmothers (F1), and a flowchart of the selection of participants across the three generations is presented in Supplementary Fig. S1. The baseline characteristics according to lifestyle of grandmothers during pregnancy are shown in Table 2. Grandmothers with a higher pregnancy lifestyle score were more likely to have a college education than those with a lower score. However, grand-maternal pregnancy lifestyle score was not associated with gestational age at delivery, age at birth, or pre-pregnancy BMI.

**Table 2:** Baseline characteristics of grandmothers (F1) by their lifestyle score during pregnancy**.**

Characteristics	Group 1 Score range: ≤ 7	Group 2 Score range: 8	Group 3 Score range: 9	Group 4 Score range: 10–12
Grandmother-child dyads	3,630	2,666	2,897	4,808
Gestational age at delivery, week	38.1 (2.1)	38.2 (1.9)	38.4 (1.9)	38.3 (1.8)
Age at birth, year	26.6 (5.2)	26.4 (5.0)	26.4 (4.8)	26.7 (4.9)
Pre-pregnancy BMI, kg/m^2^	21.2 (2.9)	21.3 (2.7)	21.1 (2.3)	21.4 (2.5)
College education, %	35	38	41	44
**Weight gain, %**				
Not adhering to the IOM guidelines	83	54	41	9
Adhering to the IOM guidelines	17	47	59	91
**Diet quality, %**				
Low	61	45	28	9
Medium	30	30	33	27
High	9	26	40	64
**Physical activity, %**				
Intense	50	38	20	10
Low	39	39	56	43
Moderate	10	22	24	48
**Smoking, %**				
Severe	22	9	4	1
Moderate	26	22	17	11
Never smoked	53	69	79	88

Plus-minus values are means ± SD.

*BMI* body mass index, *IOM* institute of medicine.

Compared to the individuals whose grandmothers had the least healthy lifestyle during pregnancy, individuals whose grandmothers had the most healthy lifestyle had 0.17 (95% CI 0.01, 0.33; P for trend = 0.05) kg/m^2^ lower BMI and 7% (95% CI 2%, 12%; P for trend = 0.001) lower risk of overweight/obesity in the offspring during adolescence and young adulthood (Table 3). The associations of grand-maternal lifestyle score with BMI and risk of overweight/obesity did not change using unadjusted and multivariate-adjusted models or additionally adjusting for F3 sex and age at BMI measurement. Moreover, the inverse associations between grand-maternal lifestyle and BMI and risk of overweight or obesity of offspring were not modified by grand-maternal pre-pregnancy BMI, offspring age, or offspring gender (P values for interaction > 0.05) (Supplementary Table S1). We conducted sensitivity analysis by creating grand-maternal lifestyle score using mean value of the four lifestyle factors, and the findings remained unchanged for offspring BMI (effect size in trend analysis − 0.06; 95% CI − 0.13, 0.01; P value: 0.09) and risk of overweight (RR in trend analysis 0.96; 95% CI 0.94, 0.99; P value: 0.003).

**Table 3:** Associations of grand-maternal lifestyle score (F1) during pregnancy with body mass index and risk of overweight of the offspring (F3).

	Group 1 Score range: ≤ 7	Group 2 Score range: 8	Group 3 Score range: 9	Group 4 Score range: 10–12	Effect size per category increment	P for trend
**Body mass index (kg/m**^**2**^**)**						
Unadjusted	Ref	− 0.21 (− 0.39, − 0.03)	− 0.29 (− 0.46, − 0.11)	− 0.27 (− 0.43, − 0.12)	− 0.09 (− 0.14, − 0.04)	0.001
Multivariable-adjusted model 1	Ref	− 0.15 (− 0.33, 0.03)	− 0.20 (− 0.38, − 0.02)	− 0.17 (− 0.33, − 0.01)	− 0.05 (− 0.10, 0.01)	0.05
Multivariable-adjusted model 2	Ref	− 0.18 (− 0.36, − 0.01)	− 0.21 (− 0.38, − 0.04)	− 0.19 (− 0.34, − 0.03)	− 0.05 (− 0.10, 0.00)	0.03
**Risk of overweight/obesity**						
Cases/participants	1,530/3,630	1,069/2,666	1,097/2,897	1,820/4,808		
Unadjusted	1.00	0.96 (0.90, 1.02)	0.91 (0.85, 0.97)	0.90 (0.85, 0.96)	0.97 (0.95, 0.98)	< 0.001
Multivariable-adjusted model 1	1.00	0.97 (0.91, 1.03)	0.93 (0.87, 0.99)	0.93 (0.88, 0.98)	0.97 (0.96, 0.99)	0.001

Generalized estimation equation (GEE) was used to account for within-family correlation of siblings and repeated measures of body mass index (BMI). Model 1 adjusted for grandmothers’ pre-pregnancy BMI (< 25 kg/m^2^, 25–30 kg/m^2^, ≥ 30 kg/m^2^), gestational age (< 38 weeks, 38–42 weeks, > 42 weeks), age at birth (quartiles), and education (middle school, high school, college). Model 2 additionally adjusted for F3 sex and F3 age at each measurement of BMI.

When each component of grand-maternal lifestyle was separately examined, smoking and gestational weight gain were independently associated with grandchild BMI or risk of overweight/obesity (Table 4). Comparing to grandmothers who smoked severely, BMI was 0.27 (95% CI 0.05, 0.49) kg/m^2^ lower in the offspring for grandmothers who were never smokers. Comparing to grandmothers with gestational weight gain outside of IOM guidelines, risk of overweight/obesity was 0.95 (95% CI 0.91, 0.99) in the offspring for grandmothers with healthy gestational weight gain. We further created the grand-maternal lifestyle score without gestational weight gain, and found that this score remained to be significantly associated with lower risk of overweight in the offspring (P for trend = 0.03).

**Table 4:** Associations of grand-maternal lifestyle factors (F1) during pregnancy with body mass index and risk of overweight of the offspring (F3).

Weight gain	Not adhering to the IOM guidelines		Adhering to the IOM guidelines	P for trend
**BMI (kg/m**^**2**^**)**				
Multivariate-adjusted	Ref		− 0.07 (− 0.20, 0.06)	0.24
**Risk of overweight/obesity**				
Cases/participants	1994/4,902		2,914/7,597	
Multivariate-adjusted	1.00		0.95 (0.91, 1.00)	0.03

Smoking	Severe	Moderate	Never smoked	
**BMI (kg/m**^**2**^**)**				
Multivariate-adjusted	Ref	− 0.27 (− 0.52, − 0.02)	− 0.27 (− 0.49, − 0.05)	0.15
**Risk of overweight/obesity**				
Cases/participants	501/1,198	1,011/2,554	4,004/10,249	
Multivariate-adjusted	1.00	0.97 (0.89, 1.06)	0.94 (0.87, 1.02)	0.11

Diet quality score	Low	Medium	High	
**BMI (kg/m**^**2**^**)**				
Multivariate-adjusted	Ref	− 0.14 (− 0.29, 0.01)	− 0.04 (− 0.19, 0.10)	0.60
**Risk of overweight/obesity**				
Cases/participants	1892/4,581	1592/4,084	1977/5,196	
Multivariate-adjusted	1.00	0.96 (0.91, 1.02)	0.95 (0.90, 1.01)	0.22

Physical activity	Low	Intense	Moderate	
**BMI (kg/m**^**2**^**)**				
Multivariate-adjusted	Ref	0.09 (− 0.06, 0.23)	− 0.01 (− 0.17, 0.15)	0.87
**Risk of overweight/obesity**				
Cases/participants	1525/3,875	2,473/6,136	1512/3,970	
Multivariate-adjusted	1.00	1.05 (1.00, 1.11)	0.98 (0.92, 1.04)	0.51

Generalized estimation equation was used to account for within-family correlation of siblings and repeated measures of body mass index (BMI). Model adjusted for grandmothers’ pre-pregnancy BMI (< 25 kg/m^2^, 25–30 kg/m^2^, ≥ 30 kg/m^2^), gestational age (< 38 weeks, 38–42 weeks, > 42 weeks), age at birth (quartiles), and education (middle school, high school, college). Weight gain during pregnancy (two categories), smoking (three categories), diet quality score (tertiles), and physical activity (tertiles) and were mutually adjusted for each other.

*IOM* Institute of Medicine, *BMI* body mass index.

**Figure 1:**
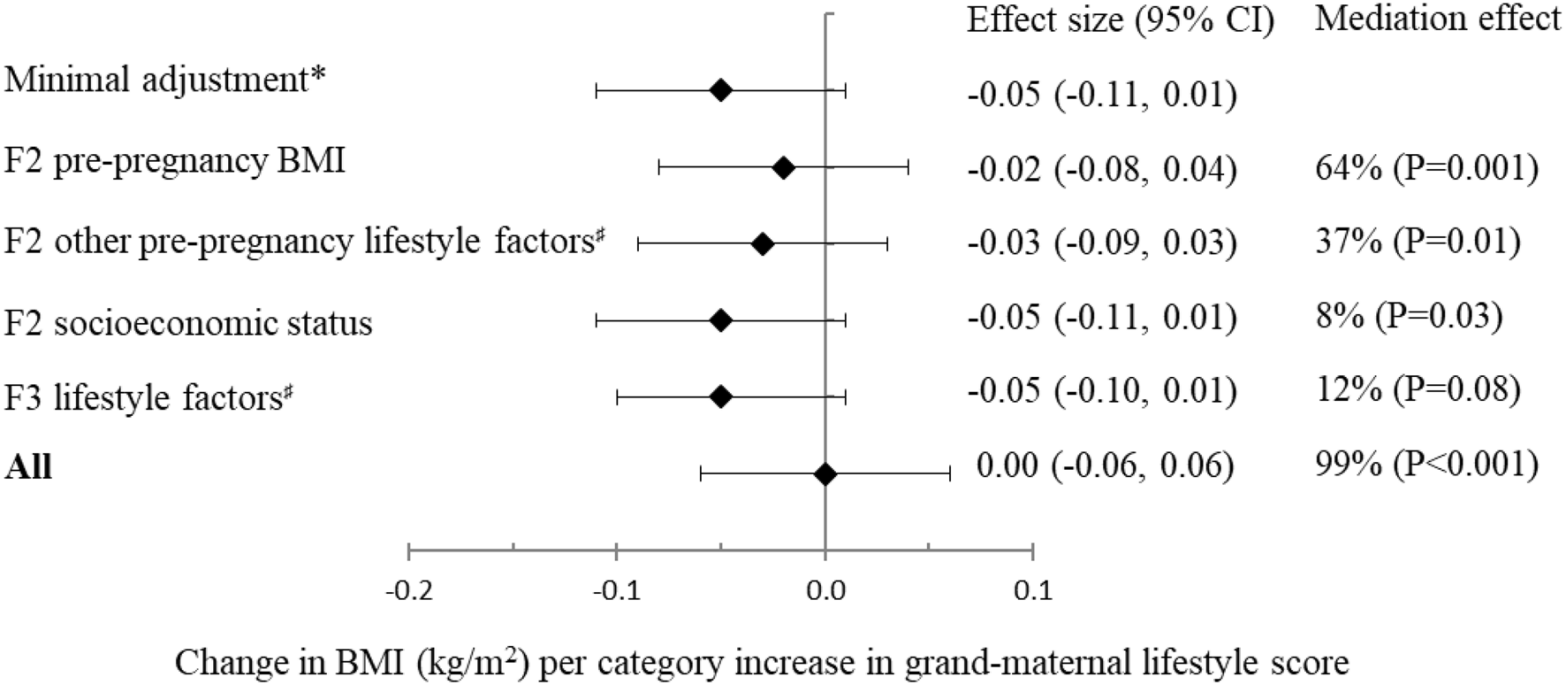
Mediation analysis for the associations between grand-maternal lifestyle score (F1) during pregnancy and body mass index of the offspring (F3). *Multivariate generalized estimation equation (GEE) adjusted for grandmothers’ pre-pregnancy BMI (< 25 kg/m^2^, 25–30 kg/m^2^, ≥ 30 kg/m^2^), gestational age (< 38 weeks, 38–42 weeks, > 42 weeks), age at birth (quartiles), and education (middle school, high school, college). ^♯^Lifestyle factors included physical activity, diet quality score, and smoking.

We examined correlations of grand-maternal lifestyle with lifestyle factors in F2 and F3 participants, and found that a healthier grand-maternal lifestyle was significantly associated with lower BMI and healthier lifestyle in F2 and higher diet quality in F3 (Supplementary Table S2). Therefore, we conducted mediation analysis to examine the percentage of inter-generational association that can be explained by lifestyle factors in F2 and F3. The inverse associations between grand-maternal lifestyle and BMI in offspring were mainly mediated by maternal pre-pregnancy BMI (mediation effect: 64%; P value = 0.001) (Fig. 1). Overall, maternal BMI along with other lifestyle factors and socioeconomic status in the second and third generations accounted for all of the inter-generational association for offspring BMI (mediation effect: 99%; P value < 0.001).

Given that only limited number of mothers and children of NHS-II participated in the Nurses’ Mother Cohort and GUTS, we tested whether missing data in F1 and F3 generations resulted in selection bias. By using a Chi-square test, we found that grandmaternal participation in the Nurses’ Mother Cohort was positively related to grandchildren participation in the GUTS (P < 0.001). We further found that individual whose grandmother had a healthier lifestyle was more likely to participate in the GUTS (P < 0.001 using Chi-square test). These findings indicated that missing data in F1 and F3 generations might cause selection bias. Thus, to account for selection bias due to censoring, we applied inverse probability weighting (IPW) to examine associations between grand-maternal lifestyle and offspring BMI, using inverse probability of being enrolled in GUTS within each category of grand-maternal lifestyle score as weights. We found that associations between grand-maternal lifestyle and offspring BMI remained the same (effect size in trend analysis − 0.05; 95% CI − 0.10, − 0.00; P value: 0.04).

As pregnancy status affects individual’s BMI, we ascertained pregnancy status in GUTS I, which was repeatedly asked in self-reported questionnaires. The incidence of being pregnant was 0.2%, 0.4%, 0.8%, 1.3%, 0.8%, and 1.5% in 2001, 2003, 2005, 2007, 2010, and 2013, respectively. We conducted sensitivity analysis by excluding body weight reported one year before and after pregnancy, and found that the associations of grand-maternal lifestyle with BMI (effect size in trend analysis − 0.05; 95% CI − 0.10, 0.00; P value: 0.05) and risk of overweight (RR in trend analysis: 0.97; 95% CI 0.95, 0.99; P value: 0.009) in the offspring remained unchanged.

## Discussion

We used data from 14,001 grandmother–mother–child triads comprised of participants of two ongoing prospective cohort studies to evaluate the role of lifestyle factors during pregnancy with weight status of grandchildren. We found that a healthy lifestyle of grandmothers during pregnancy including adequate weight gain, a high-quality diet, moderate amount of physical activity, and no smoking was associated with lower BMI and lower risk of overweight in the offspring. Of these four factors, adequate gestational weight gain and avoidance of tobacco smoking had the greatest impact.

### Maternal lifestyle and body weight in the offspring

Literature about early life origins of obesity has focused on transgenerational effect of maternal lifestyle with risk of obesity in the offspring, and strong evidence suggested that excess weight gain and smoking during pregnancy were risk factors of obesity in the offspring. For example, studies showed that gestational weight gain below or above the Institution of Medicine (IOM) guidelines was associated with higher birth weight and higher risk of obesity in the offspring^7,8,17^; maternal smoking during pregnancy was associated with higher risk of obesity in children in a dose response manner regardless of the time window of the exposure^9,10^. With regard to diet and physical activity, two recent meta-analyses of RCTs on diet and physical activity based interventions in pregnancy found that a healthy diet and adequate amount of exercise during pregnancy were beneficial for offspring mainly by preventing excessive gestational weight gain^12,13^. Studies evaluating an overall healthier pre-pregnancy lifestyle consisting of multiple lifestyle practices found that maternal adherence to such a lifestyle was associated with lower risk of obesity in the offspring^14,15^. Despite these existing findings, few studies have focused on the overall maternal lifestyle during pregnancy and extended the follow-up period to examine the association with risk of obesity in the third generation. By merging the Nurses’ Mothers’ Study, the NHSII, and GUTS, our study was the first one to show that the beneficial effect of a healthy lifestyle during pregnancy on body weight persisted to the third generation.

### Mediators between grand-maternal lifestyle and body weight in the offspring

We conducted mediation analysis to understand why a healthy grand-maternal lifestyle during pregnancy was associated with lower BMI, and found that F2 and F3 lifestyle factors together with families’ socioeconomic status largely explained the association. Particularly, the F2 maternal pre-pregnancy BMI was the predominant mediator between grand-maternal lifestyle and offspring BMI. This indicated that a less healthy grand-maternal lifestyle during pregnancy was associated with higher maternal BMI, which resulted in higher BMI in the third generation. This finding is supported by evidence from observational studies as mentioned above. Moreover, studies indicated that the observational associations between maternal BMI and body weight in the offspring may be causal. By adopting a sibling design, one study showed that maternal weight gain during pregnancy increased birthweight independently of genetic factors^28^. In a Mendelian randomization study involving 30,487 women, genetically elevated maternal BMI was potentially causally associated with higher offspring birth weight^29^. It is plausible that the effect of maternal BMI and gestational weight gain on offspring body weight can be inherited to the third generation. Besides maternal BMI as potential mediator, we found that other lifestyle factors including diet, physical activity, and smoking in the second and third generation partially explained the association. The reason might be that mothers’ lifestyle and behaviors could shape their children’s own lifestyle and diet and subsequently modulated offspring’s obesity risk^30,31^*.* Studies showed that family lifestyle and household routines were associated with risk of obesity in the offspring^32,33^. In addition, socioeconomic status has been identified as a risk factor of obesity, and children born in low socioeconomic families were associated with higher risk of obesity^34,35^.

### Molecular mechanisms between maternal lifestyle and body weight in offspring

The biological mechanisms of how smoking during pregnancy and maternal BMI affected BMI in the offspring have been investigated, and epigenetic processes may play an important role. Previous research suggested that changes in epigenetic processes such as alterations in DNA methylation might play a role^36^. Epigenome-wide association studies showed that maternal smoking during pregnancy was associated with differences in DNA methylation in newborns, and this association persisted until adolescence^37,38^. Three cohort studies showed that maternal obesity was associated with methylation of DNA in cord blood at birth^39–41^, and the effect was also present at 3 year follow-up^39^. Another study compared DNA methylation among siblings born to obese mothers before and after bariatric surgery with associated weight loss, and found that the CpG sites were differentially methylated^42^. In addition to epigenetic changes, intra-uterine programming mechanisms might also be involved. Maternal obesity was associated with increased pre-pregnancy insulin resistance and hyperinsulinemia, inflammation, and oxidative stress^43^. Maternal pathophysiological status may contribute to early placental and fetal dysfunction in the offspring that may persist to adulthood, including changes in function of adipose tissue, appetite regulation, and energy metabolism^44^.

It is plausible that grand-maternal lifestyle affects maternal BMI through alterations in DNA methylation or early fetal dysfunction. And maternal BMI was subsequentially related to risk of obesity in the offspring. For future research, it would be worthwhile to examine whether the physiological changes affect the third generation.

### Strengths and limitations of the study

One main strength of our study is the establishment of this unique three generation design comprised of the Nurses’ Mother Cohort and two other large ongoing cohorts. Nurses’ mothers provided detailed information on lifestyles during pregnancy by questionnaire, which enabled us to examine whether grand-maternal lifestyle associated with risk of obesity across three generation. Moreover, we obtained data on F2 and F3 lifestyle factors with multiple repeated measures during follow-up period, allowing us to explore the mechanism of how grand-maternal lifestyle affected risk of obesity in the offspring. Our study is not without limitations. First, gestational weight gain is genetically correlated with BMI, and study has found that some established variants associated with increased BMI were associated with higher later gestational weight gain^45^. Although our study included sibling pairs in the GUTS, we cannot control for genetic components as all siblings shared the same grand-maternal exposure during pregnancy. However, by utilizing available genome-wide genotyping data among a limited number of participants in the NHS-II (n = 1,632), we examined mediation effect of BMI genetic risk score based on 97 genetic variants between grand-maternal lifestyle and offspring BMI^46^, and no significant mediation effect was found (4.9%; 95% CI 0–96.3%; P = 0.37). Second, grand-maternal information were collected retrospectively on the lifestyle during pregnancy of their nurse daughter which occurred decades ago. The long-term memory might be subject to measurement error. However, the measurement error should be independent of the obesity status of the F3 participants, and therefore was likely to be non-differential, meaning that the measurement error was likely to add random noise to the associations rather than systematic errors. Moreover, although non-differential measurement error of the exposure attenuates associations towards null, we still found an inverse association between grand-maternal lifestyle and offspring BMI. Third, the BMI of GUTS participants were self-reported, which might be prone to measurement error. However, the accuracy of self-reported body weight has been examined in validation studies among adolescents, and their self-reported weight were shown to be highly reliable^21–23^. Fourth, the NHSII are predominantly white health professionals which may be the reason that we did not observe strong mediation effect by socioeconomic status and also limit the generalizability of the findings. However, their occupational status is a distinct advantage that allows us to collect high-quality data using self-reported questionnaires.

In conclusion, we found that greater adherence to smoking, diet, physical activity and weight gain recommendations during pregnancy was associated with lower BMI and lower risk of overweight among grandchildren. Some of this relation was explained by maternal BMI and increased adherence to healthy lifestyles among mothers suggesting that the overall association may be a reflection of both shared environment and behaviors across generations and transgenerational effects due to the effects of the lifestyle factors examined on the fetal ovary. Regardless of the specific mechanisms, whether biological or behavioral, our findings suggest that the benefits of adherence to weight gain and lifestyle recommendations during pregnancy is likely to have a positive health impact across generations.

## Supplementary information

Supplementary Information.

## Data Availability

No additional data available.
