# Supplementary material for: Grand-maternal lifestyle during pregnancy and body mass index in adolescence and young adulthood: an intergenerational cohort study
Source: Sci Rep. 2020 Sep 2;10:14432. doi: 10.1038/s41598-020-71461-5 (PMC7468235; doi:10.1038/s41598-020-71461-5)
Supplement: Supplementary file 1 — Supplementary Information. [file 41598_2020_71461_MOESM1_ESM.docx]

**Grand-maternal Lifestyle During Pregnancy and Body Mass Index in adolescence and young adulthood: an Intergenerational Cohort Study**

Ming Ding ^1^, Susanne Strohmaier ^2,3^, Eva Schernhammer ^3,4^, Changzheng Yuan ^1,5^, Qi Sun ^1,2^, Karin B. Michels ^6^, Rulla Tamimi ^7,8^, Jorge E. Chavarro ^1,4,8^

**Figure S1. Flowchart of the selection of participants across the three generations.**

**F1: Nurses’ Mother Cohort:** Grand-maternal lifestyle factors during pregnancy were main exposures.

Nurses’ Mother Cohort initially included 35,794 participants, and our study included 9157 participants born to 14,001 grand-children enrolled in GUTS.

**F2: Nurses’ Health Study-II:** Pre-pregnancy lifestyle factors were treated as potential mediators.

NHS-II initially included 116,430 participants, and our study included 9157 participants whose mother enrolled in the Nurses’ Mother cohort and whose children participated in the GUTS.

**F3: Growing Up Today Study:** Body mass index and risk of overweight were main outcome.

Initially, 16,882 individuals in GUTS I and 10,923 individuals in GUTS II returned a baseline questionnaire (n=27,805)

We excluded individuals with no grand-maternal information (n=13,773); and grandmothers who had missing data on ≥2 lifestyle factors during pregnancy (n=31)

Finally, 14,001 GUTS participants were included in our study, who was born to 9157 mothers in the NHS-II and grandmothers in the Nurses’ Mother cohort.

**Table S1. Stratified analysis for the associations between grand-maternal lifestyle score (F1) during pregnancy and anthropometric characteristics of offspring (F3).**

|  | Change in BMI per category increment in lifestyle score | P for interaction |  | Risk of overweight/obesity per category increment in lifestyle score | P for interaction |
| --- | --- | --- | --- | --- | --- |
| **F1 pre-pregnancy BMI** |  |  | **F1 pre-pregnancy BMI** |  |  |
| Normal | -0.06 (-0.12, -0.01) | 0.18 | Normal | 1.00 (0.94, 1.05) | 0.34 |
| Overweight/obesity | 0.07 (-0.14, 0.27) |  | Overweight/obesity | 0.97 (0.95, 0.99) |  |
| **F3 age (time-varying)** |  |  | **F3 age (baseline)** |  |  |
| ≤18 years | -0.05 (-0.10, -0.00) | 0.58 | ≤18 years | 0.97 (0.96, 0.99) | NA |
| >18 years | -0.05 (-0.12, 0.03) |  | >18 years | NA |  |
| **F3 gender** |  |  | **F3 gender** |  |  |
| Female | -0.05 (-0.12, 0.02) | 0.50 | Female | 0.99 (0.96, 1.01) | 0.17 |
| Male | -0.05 (-0.13, 0.03) |  | Male | 0.97 (0.94, 0.99) |  |

Generalized estimation equation (GEE) was used to account for within-family correlation of siblings and repeated measures of body mass index (BMI). Multivariate GEE adjusted for grandmothers’ pre-pregnancy BMI (<25kg/m^2^, 25-30 kg/m^2^, ≥30 kg/m^2^), gestational age (< 38 weeks, 38-42 weeks, >42 weeks), age at birth (quartiles), and education (middle school, high school, college).

**Table S2. Associations of grand-maternal lifestyle (F1) with lifestyle factors in the mothers (F2) and offspring (F3).**

| Characteristics | Group 1  score range: 4-7 | Group 2  score range: 8 | Group 3  score range: 9 | Group 4  score range: 10-12 | P value for Chi-Square test |
| --- | --- | --- | --- | --- | --- |
|  |  | Maternal status | |  |  |
| Pre-pregnancy BMI, kg/m^2^ |  |  |  |  |  |
| Normal | 74 | 78 | 76 | 77 |  |
| Overweight | 18 | 15 | 17 | 16 |  |
| Obese | 8 | 7 | 7 | 6 | 0.004 |
| Diet quality, % |  |  |  |  |  |
| Low | 38 | 34 | 32 | 31 |  |
| Medium | 31 | 34 | 32 | 36 |  |
| High | 32 | 32 | 36 | 33 | <0.001 |
| Physical activity, % |  |  |  |  |  |
| Low | 36 | 32 | 32 | 32 |  |
| Moderate | 34 | 34 | 33  35 | 33 |  |
| Intense | 30 | 34 |  | 34 | <0.001 |
| Smoking, % |  |  |  |  |  |
| Never smoker | 72 | 72 | 72 | 73 |  |
| Past or current smoker | 28 | 28 | 28 | 27 | 0.83 |
|  |  | Grand-maternal status | |  |  |
| Diet quality, % |  |  |  |  |  |
| Low | 30 | 30 | 28 | 27 |  |
| Medium | 38 | 38 | 38 | 39 |  |
| High | 31 | 31 | 34 | 35 | <0.001 |
| Physical activity, % |  |  |  |  |  |
| Low | 31 | 31 | 31 | 31 |  |
| Moderate | 40 | 40 | 39 | 41 |  |
| Intense | 29 | 30 | 30 | 29 | 0.89 |
| Smoking, % |  |  |  |  |  |
| Never smoker | 89 | 89 | 90 | 88 |  |
| Past or current smoker | 11 | 11 | 10 | 12 | 0.06 |
